# *Epidermodysplasia verruciformis* human papillomavirus types and carcinoma of the conjunctiva: a pilot study

**DOI:** 10.1038/sj.bjc.6601743

**Published:** 2004-04-06

**Authors:** C Ateenyi-Agaba, E Weiderpass, A Smet, W Dong, M Dai, B Kahwa, H Wabinga, E Katongole-Mbidde, S Franceschi, M Tommasino

**Affiliations:** 1 Department of Ophthalmology, Makerere University, PO Box 7072, Kampala, Uganda; 2 International Agency for Research on Cancer, Unit of Field and Intervention Studies, World Health Organization, 150 Cours Albert Thomas, 69372 Lyon, France; 3 Department of Epidemiology and Biostatistics, Karolinska Institutet, PO Box 281, S-171 77 Stockholm, Sweden; 4 Jinja Hospital, PO Box 2004, Jinja, Uganda; 5 Department of Pathology, Makerere University, PO Box 7072, Kampala, Uganda; 6 Uganda Cancer Institute, PO Box 7051, Kampala, Uganda

**Keywords:** squamous-cell carcinoma of the conjunctiva, human papillomavirus (HPV), PCR-based assays, *Epidermodysplasia verruciformis* HPV types, Uganda

## Abstract

A total of 21 squamous-cell carcinoma of the conjunctiva (SCC) and 22 control subjects had conjunctival samples tested for human papillomavirus (HPV) types using PCR-based assays. *Epidermodysplasia verruciformis* HPV types were found in 86% of SCC cases and 36% of control subjects (Odds ratio=12.0), suggesting a role of HPVs in the aetiology of SCC.

Squamous-cell carcinoma of the eye conjunctiva (SCC) is a rare tumour associated with heavy sun exposure, which has greatly increased in some Sub-Saharan African countries, coincident with the spread of HIV (Newton, 1996). An increased risk of SCC has been reported in HIV-positive individuals in the United States (Goedert and Cote, 1995). The link with immune impairment suggests that infectious agents such as epithelio-tropic human papillomaviruses (HPVs) may be involved in the aetiology of SCC.

HPVs comprise mucosal and cutaneous types, the latter being found in skin carcinomas from patients with *Epidermodysplasia verruciformis (EV)* (zur Hausen, 2000). A subgroup of high-risk mucosal HPV types, notably HPV 16, 18 and 45, are the cause of cervical carcinoma and of a subset of ano-genital and head and neck carcinomas (zur Hausen, 2000). Low-risk HPV types 6 and 11 have been detected in benign papillomas of the conjunctiva, while studies of SCC have produced inconsistent results. A few studies found HPV types 16 and 18 DNA in SCC tissue biopsies, while others did not (Newton, 1996). Squamous-cell carcinoma of the conjunctiva risk was not associated with antibodies against HPV16, 18 and 45 in a case–control study in Uganda (Newton *et al*, 2002).

## METHODS

Between March 2000 and January 2001, we conducted a pilot case–control study on 21 SCC cases (mean age: 33.5, range 23–49) and 22 age frequency-matched control patients with benign conjunctival lesions (mean age 33.8, range 14–55) in New Mulago Eye Department, Kampala, Uganda, where SCC has become the most common ocular malignancy. This aimed to assess the feasibility of a larger case–control study in that location, and to test methods.

All patients signed an informed consent and the study was approved by the local Ethical Committee. In total, 10 controls had pterygium, seven pingueculum, four solar keratosis and one pigmented naevus. Tissue specimens from SCC cases and control patients were collected, histologically confirmed, and deep-frozen before shipment to the International Agency for Research on Cancer (IARC), Lyon, France, for HPV testing. To minimise the risk of contamination, the collection of the specimens was performed at different days using disposable material. DNA was extracted from each sample in an IARC laboratory that is exclusively used for this purpose. To monitor the possible occurrence of cross contamination between the different specimens during the extraction of DNA, tubes containing distilled water (one every 10 specimens) were included.

HPV detection was performed by means of polymerase chain reaction (PCR) assays using different sets of primers (Chen *et al*, 1994; Berkhout *et al*, 1995; Jacobs *et al*, 1995; Forslund *et al*, 1999, Harwood *et al*, 1999; Caldeira *et al*, 2003). Laboratory personnel were blinded to the histological diagnosis of each patient.

The PCR mixture was prepared in a special laboratory (pre-PCR laboratory) in sterile conditions, while the PCR and analysis of products were performed in different laboratories. As in the DNA extraction protocol, negative controls were included in the different PCRs. Products obtained with all type-specific primers were also tested by Southern blotting. All analyses, including cellular DNA extraction, PCR, and Southern blotting, were performed in duplicate using consecutive sections of frozen specimens.

## RESULTS

We first determined the presence of the mucosal HPV types using the general primers (GP) 5+/6+, GP1/GP2 and specific primers for HPV16, HPV18 and HPV45. No mucosal types were found in either cases or controls, but in one SCC case (patient number 21) we detected, by means of GP1/GP2 primers, a novel HPV type closely related to EV HPV type RTRX7 (Table 1

**Table 1:** Detection of DNA of HPV in biopsies from 21 cases of carcinoma of the conjunctiva and 22 control patients. Kampala, Uganda, 2000–2001

		**PCR primers^a^**						
**Type of lesion (patient identification number)**	**Age**	**GP1/GP2**	**FAP59/FAP64**	**CP62/69 EN3F/EN3R**	**CP62/69 EN1F/EN1R**	**CP65/CP70 CP66/CP69**	**HPV38 E6**	**Summary results of EV HPV positive^b^**
Carcinoma (1, 5, 8, 9, 10, 11, 12, 13, 16, 19, 20)	25; 30; 26; 42; 34; 24; 30; 39; 48; 30; 43	−	−	−	−	−	+	+
Carcinoma (2, 15, 17)	30; 23; 35	−	−	−	−	−	−	−
Carcinoma (3)	25	−	−	HPV12 related^c^	HPV8 related^c^	−	+	+
Carcinoma (4)	39	−	−	−	HPV24^c^	+	HPV38^c^	+
Carcinoma *in situ* (6)	29	−	−	−	−	+	HPV38^c^	+
Carcinoma (7)	29	−	−	−	HPV14^c^	−	+	+
Carcinoma (14)	49	−	−	−	−	+	+	+
Carcinoma (18)	37	−	−	HPV37^c^	HPV36^c^	+	+	+
Carcinoma (21)	36	RTRX7 related^c^	−	−	−	−	−	+
Pterygium (22)	33	−	HPV11^c^	−	−	−	+	+
Pterigium (23)	19	−	HPV11^c^	−	−	−	−	−
Pterygium (24)	45	−	−	−	−	−	HPV38^c^	+
Pterygium (25, 27, 28, 29, 30)	48; 37; 50; 29; 33	−	−	−	−	−	−	−
Pingueculum								
(32, 33, 36, 37, 38)	27; 48; 28; 27; 30	−	−	−	−	−	−	−
Pterygium (26, 31)	29; 26	−	−	−	−	−	+	+
Pingueculum (34, 35)	NA; 32	−	−	−	−	−	+	+
Solar keratosis (39, 41; 42)	40; NA; 25	−	−	−	−	−	−	−
Solar keratosis (40)	55	−	−	−	−	−	+	+
Pigmented naevi (43)	14	−	−	−	HPV5^c^	HPV38^c^	+	+

a The PCR data obtained with GP5+/GP6+, HPV10, HPV16, HPV18, HPV20, HPV22, HPV23 and HPV45 primers were not included in the table since no positive cases were detected.

b Positive sign indicates that HPV was detected in any of previous columns in the table.

c The HPV type was determined by direct DNA sequence of the PCR product. NA=not available.

). To search for other EV HPV types, we used the FAP59/64 primers, which were designed from two relatively conserved regions present in the L1 gene of most HPV types, including the cutaneous ones. The low-risk mucosal HPV type 11 was detected in two controls with pterygium (patient numbers 22 and 23; Table 1).

We then followed three highly sensitive nested PCR protocols for detecting EV HPV types: CP62/69-EN3F/EN3R; CP62/69-EN1F/EN1R; and CP66/CP69-CP65/CP70 (Berkhout *et al*, 1995; Harwood *et al*, 1999). We found additional EV HPV types in six cases (patient numbers 3, 4, 6, 7, 14, 18) and one control (patient number 43). In the majority of the HPV positive subjects (patient numbers 3, 4, 7, 18 and 43) we were able to directly sequence the PCR product and identify the HPV type, including one each for HPV5, HPV14, HPV24, HPV36, HPV37 and HPV38, in addition to two novel types closely related to HPV8 and HPV12. In two cancer cases (patient numbers 3 and 18) and in one control (patient number 43) we detected multiple HPV infections using these nested PCR protocols (Table 1). The SCC patients positive for HPV 24 (patient number 4) and HPV 36 and 37 (patient number 18) were also positive with the protocol CP65/CP70–CP66/CP69, but we were not able to sequence the PCR products and identify the specific HPV type(s) involved.

Finally, we performed a PCR analysis followed by Southern blotting using specific primers for the EV HPV type 38 that we have used in a previous study on nonmelanoma skin cancer (Caldeira *et al*, 2003). These primers are approximately 50–100 fold more sensitive than the CP primers described above in detecting HPV38 (data not shown). HPV38 E6 specific PCR followed by Southern blotting showed a positive signal in 17 SCC cases and eight controls. Only in two SCC cases (patient numbers 4 and 6) and one control (patient number 24) were we able to demonstrate the presence of HPV38 by DNA sequencing. One case (patient number 4) was positive for both HPV 38 and HPV 24. Positive signals at Southern blotting, not confirmed by DNA sequencing, are likely to correspond to the presence of HPV types closely related to HPV38 (Table 1). Testing for HPV22 and HPV23, which are closely related to HPV38, and HPV10, that is associated with benign skin lesions, gave negative results for all patients. The results of the different PCR protocols used in the study are summarised in the last column of Table 1. Overall, EV HPV types were found in 18 out of 21 (86%) SCC cases and in eight out of 22 (36%) controls.

SCC cases and 20 controls who had a questionnaire including sociodemographic and lifestyle information are compared in Table 2

**Table 2:** ORs and corresponding 95% CIs of carcinoma of the conjunctiva by presence of EV HPV DNA among 21 cases and 20 controls, Kampala, Uganda, 2000–2001

	**Cancer cases**	**Controls**	**OR^a^**	**(95% CI)**	**OR^b^**	**(95% CI)**
*HPV DNA*						
Absent	3	13	1		1	
Present	18	7	12.0	(1.7–84.9)	22.7	(1.7–312)
						
*Education*						
Primary or less	17	8	1			
Secondary	4	12	0.3	(0.06–1.1)		
						
*Occupation*^c^						
Indoor	5	13	1			
Outdoor	15	6	3.2	(0.7–13.8)		
						
*Sun exposure (daily hours)*^c^						
<6	10	17	1		1	
>7	11	2	6.4.	(0.8–54.2)	29.0	(0.7–1137)

a Adjusted for age and gender by means of Mantel–Haenszel procedure.

b Adjusted for EV HPV DNA or sun exposure, as appropriate.

c Some figures do not add up to the total because of missing values. EV HPV=*Epidermodysplasia verruciformis* (EV) types of human papillomavirus (HPV).

by means of age-adjusted odds ratios (OR) and corresponding 95% confidence intervals (CI). The OR for the presence of EV HPV was 12.0 (95% CI: 1.7–84.9). The prevalence of EV HPV did not vary by age group in either cases or controls. Educational level was inversely associated with SCC risk, whereas outdoor occupation and sun exposure were directly associated. On account of the small number of study patients, the corresponding confidence intervals were broad and included unity, except for EV HPV. The association between EV HPV and SCC was strengthened (OR=22.7) by adjustment for sun exposure (Table 2). We repeated all analysis in Table 2 excluding from the control group the three patients with solar keratosis from whom a questionnaire was available. These lesions have been suggested to be premalignant lesions and may dilute the associations studied. The results obtained were not materially different from the results presented in Table 2 (e.g. OR for EV HPV DNA after exclusion of solar keratosis=21.8; 95% CI=1.4–344).

## DISCUSSION

The most severe limitation of our findings is that HIV serology was not available for our study patients. Although medical records did not mention AIDS, it is likely that a few controls but a majority of our SCC cases were HIV-positive, as found in a previous study in Uganda (Newton *et al*, 2002). We cannot, therefore, rule out the possibility that immunosuppression due to HIV infection may partly or even totally explain the strong association between EV HPV and SCC in this study.

We detected in SCC cases eight different EV HPV types (HPV8, HPV12, HPV14, HPV24, HPV36, HPV37, HPV38 and RTRX7) in addition to several undetermined EV HPV types related to HPV38. HPV38 displays *in vitro* transforming properties and has been found in nonmelanomatous skin cancer (Caldeira *et al*, 2003). In three SCC cases two different EV HPV types were detected. The prevalence of EV types was significantly greater among SCC cases than among controls, although the impact of HIV infection on this finding is unknown.

The extreme heterogeneity of the cutaneous HPV family and the lack of standard GP for their detection greatly complicate the study of the relationship between the cutaneous HPV types and cancers like SCC. However, the strong association that we found between EV HPV types and SCC points to a possible aetiological role of these viruses.

## References

[bib1] Berkhout RJ, Tieben LM, Smits HL, Bavinck JN, Vermeer BJ, ter Schegget J (1995) Nested PCR approach for detection and typing of *Epidermodysplasia verruciformis*-associated human papillomavirus types in cutaneous cancers from renal transplant recipients. J Clin Microbiol 33: 690–695 7751378 10.1128/jcm.33.3.690-695.1995 PMC228015

[bib2] Caldeira S, Zehbe I, Accardi R, Malanchi I, Dong W, Giarre M, deVilliers E-M, Filotico R, Boukamp P, Tommasino M (2003) The E6 and E7 proteins of cutaneous human papillomavirus type 38 display transforming properties. J Virol 77: 2195–2206 12525654 10.1128/JVI.77.3.2195-2206.2003 PMC140944

[bib3] Chen B, Yin H, Dhurandhar N (1994) Detection of human papillomavirus DNA in esophageal squamous cell carcinomas by the polymerase chain reaction using general consensus primers. Hum Pathol 25: 920–923 8088768 10.1016/0046-8177(94)90012-4

[bib4] Forslund O, Antonsson A, Nordin P, Stenquist B, Hansson BG (1999) A broad range of human papilloma virus types detected with a general PCR method suitable for analysis of cutaneous tumours and normal skin. J Gen Virol 80: 2437–2443 10501499 10.1099/0022-1317-80-9-2437

[bib5] Goedert JJ, Cote TR (1995) Conjunctival malignant disease with AIDS in USA. Lancet 346: 257–258 10.1016/s0140-6736(95)91309-2 7616836

[bib6] Harwood CA, Spink PJ, Surentheran T, Leigh IM, de Villiers EM, McGregor JM, Proby CM, Breuer J (1999) Degenerate and nested PCR: a highly sensitive and specific method for detection of human papillomavirus infection in cutaneous warts. J Clin Microbiol 37: 3545–3555 10523550 10.1128/jcm.37.11.3545-3555.1999 PMC85688

[bib7] Jacobs MV, de Roda Husman AM, van den Brule AJ, Snijders PJ, Meijer CJ, Walboomers JM (1995) Group-specific differentiation between high- and low-risk human papillomavirus genotypes by general primer-mediated PCR and two cocktails of oligonucleotide probes. J Clin Microbiol 33: 901–905 7790457 10.1128/jcm.33.4.901-905.1995 PMC228064

[bib8] Newton R (1996) A review of the aetiology of squamous cell carcinoma of the conjunctiva. Br J Cancer 74: 1511–1513 8932327 10.1038/bjc.1996.581 PMC2074846

[bib9] Newton R, Ziegler J, Ateenyi-Agaba C, Bousarghin L, Casabonne D, Beral V, Mbidde E, Carpenter L, Reeves G, Parkin DM, Wabinga H, Mbulaiteye S, Jaffe H, Bourboulia D, Boshoff C, Touze A, Coursaget P, Uganda Kapsi's Sarcoma Study Group (2002) The epidemiology of conjunctival squamous cell carcinoma in Uganda. Br J Cancer 87: 301–308 12177799 10.1038/sj.bjc.6600451 PMC2364227

[bib10] zur Hausen H (2000) Papillomavirus causing cancer: evasion from host-cell control in early events in carcinogenesis. J Natl Cancer Inst 92: 690–698 10793105 10.1093/jnci/92.9.690

